# Enough with simplifying: “eat less and move more”: at what point are we with the treatment of excess weight in paediatrics?

**DOI:** 10.1186/s13052-024-01676-z

**Published:** 2024-05-23

**Authors:** Rita Tanas, Giovanni Corsello, Riccardo Lera, Maria Marsella, Sergio Bernasconi

**Affiliations:** 1 Pediatric Department, Ferrara, Italy; 2 Pediatric Department G. D’Alessandro, University of Palermo, Palermo, Italy; 3 Pediatric Department, Azienda Ospedaliera, Alessandria, Italy; 4 Pediatric Unit, Maternal-Pediatric Department, San G. Moscati Hospital, Avellino, Italy; 5 Microbiome Research Hub, University of Parma, Parma, Italy; 6 Via Fornace 17, Ferrara, 44123 Italy

**Keywords:** Obesity, Weight stigma, Weight management

## Abstract

**Background:**

For years politics and healthcare, faced with the progressive increase in the prevalence of overweight and obesity in childhood, have wondered how to stem it and reduce its consequences on health without finding a valid, effective and applicable solution. Many studies have been written initially on what to prescribe, then on why not to prescribe and how to approach people in a new and more effective way to improve their behaviors, considered the main cause of excess weight. Over the last twenty years it has been highlighted that no diet or physical exercise is truly effective and not even global changes in lifestyle guarantee the large weight reductions traditionally expected, despite offering significant health advantages. A new approach is necessary and we must begin by working on ourselves.

**Main body:**

We examined literature on weight stigma and considered expert opinions, as well as feedback from parents/caregivers and patients. Literature on stigma has grown enormously in recent years, and finally considers the opinion of parents and patients. By interviewing patients with obesity, it was discovered that very often healthcare workers do not communicate the diagnosis and, if they do, they have a blaming attitude, holding patients responsible for their weight. Furthermore, when these people become aware of their obesity and seek treatment, they do not find adequate professionals and centers. Failure was mostly due to the enormous burden of obesity stigma and discrimination which, especially in children and young people, encourages internalization of the problem and takes away their self-efficacy, desire and ability to take care of themselves.

**Conclusions:**

New actions are needed to change all this. We propose “Training, Networking and Contrasting Weight Stigma”. Now that we’ve figured out where to start, we should get going. And yet, nothing is changing!

## Background

What has changed since the 2020 commentary [1]?

Four years have passed since our 2020 commentary on weight stigma, but almost nothing has changed. Despite the laws of health urgency, the prevalence of obesity continues to increase, stealing years of life and health of a large part of the world population.

## Main text

We have not yet given due value to its social determinants and adverse childhood experiences, nor have we considered the adoption in daily practice of a respectful approach, according to the principles of the motivational interview, as required by the latest US guidelines [2]. We have not yet adopted Person-first language, nor changed stigmatizing images in training. We did not move the assessment of the child with excess weight from mere Body Mass Index (BMI) zscore to evaluation of general health using the Edmonton Obesity Stage System for Pediatrics [3]. We have not changed the goal of care from weight loss to improved health. So we are preparing to deal with obesity intensively, as requested by the WHO (World Health Organization) European Region 2022 document, without the right tools and with the risk of doing more harm than good [4].

Today it is quite fashionable to talk about excess weight in families, schools and healthcare, but few actually do anything to improve the situation (Table 1).

**Table 1:** Potential interventions for obesity prevention in different settings. (Adapted from Lister et al. 2023) [5]

**Preschool and school settings**	
**Eliminate/restrict choice**	**Guide/enable choice**
• Monitor the content of packed lunches • Eliminate sugar-sweetened beverages in vending machines and replace with water • Limit chips or French fries to once a week	• Offer free fruit at break and snack times • Include bursts of physical activity during classroom time • Introduce after-school dance or sport sessions • Provide safe cycling and walking routes to school
**Community-level settings**	
Town planning policies on mobile food and beverage vans close to schools and restrictions on the number and locations of takeaway food outlets on walking journeys undertaken by children	Local youth groups run by non-governmental organizations targeted in disadvantaged areas that promote physical activity
**Public policy**	
Advertising of foods high in fats, sugar and salt and of sugary beverages during prime-time children’s television In supermarket all high-fat, high-sugar and high-salt snack foods from payment counter areas (to limit ‘pester power’)	Financial support from industries for sporting and physical activities and events for children Positive messages about healthy lifestyles from role models via social media

Already in 2020 we had underlined how it is to fight weight stigma in healthcare, but also in the other educational institutions, like schools and families [1]. Unfortunately, actions in this field are still lacking. We talk about it more and more, but it is clear that talking about it, not only doesn’t work, but can do much more harm [6, 7].

Literature on stigma has grown enormously, finally considering children and families. It has been highlighted that derision affects children and parents (association stigma) since pregnancy, negatively impacting health throughout their lives [8], and creating an obstacle to treatment [9].

If we truly wish to deal with obesity, as international organizations require, there are many things to do, such as involving political institutions, who currently do not collaborate with each other. Alongside the regulating marketing strategies aimed at children and adolescents and reducing sugar and sweeteners, based on the principle “everyone must do their part!“, as healthcare workers, we must ask ourselves “where to start”.

We propose “Training, Networking and Contrasting Weight Stigma”, magic words which must become operational.

### Training

The first move is always “training”, a journey that has already begun. But training must not only concern epidemiology, diagnosis, therapy and complications which, in the absence of early behavioural treatment, are perceived by doctors and patients as faults, diagnosed too late and therefore treated with the usual methods. Training in Italy is mainly entrusted to university structures, which offer it to students, specialists and healthcare professionals.

The change of perspective regarding obesity, with the involvement of patients in the development of therapeutic paths, however has not yet been truly adopted by anyone. Only when trainers will have shared the objective of fighting weight stigma and managing obesity as a “chronic disease”, will we be able to carry out a “new” form of training! Furthermore, according to Andragogy, the science of adult education, training must be enjoyable for the learners.

But family and hospital paediatricians and paediatric nurses are absolutely convinced that they are well prepared to cure children with obesity [10]: there is nothing new to know, apart from drugs to be used with caution in developmental age, the usual recommendations, which families already know, and the diagnostics to be prescribed. They believe that nothing can be done to treat obesity, as it is considered just “a waste of time”. Unfortunately, attention to professional and family stigma creates feelings of guilt in professionals, especially the best ones, who defend themselves by denying and shifting attention to other less problematic illnesses.

In this way training towards a change of perspective is still lacking [11]. The training courses on the treatment of obesity are carried out with old clichés: not new ways of communicating with the patient, better professional-family-child/young relationships and creating awareness to contrast weight stigma, but rather concentration on breastfeeding, weaning and complications. Usual topics that have already proven to be unsuccessful, while we know that reducing stigma and guilt, although difficult, would improve the quality of life, even if not always weight, especially in the long term.

### Fighting stigma

Obesity, like all chronic diseases, is not healable. Reversing it, when it has become structured and, above all, has become serious, is almost impossible. If it is difficult to regain a “normal” weight, as with all chronic pathologies, with treatment patients may feel better, reduce cardio-metabolic risk, improve sociability and quality of life. It is necessary to re-educate professionals starting from the definition of obesity as a complex, progressive and relapsing chronic disease with many contributing social and environmental factors (Table 2), so to reduce the sense of guilt of both patients and professionals, who must be satisfied with having improved the health of their patients, despite not being able to achieve complete healing.

**Table 2:** Multilevel Influencers and Contributors to Obesity. (adapted from Hampl et al. 2023) [2]

**A. Policy** 1. Marketing of unhealthy foods 2. Under resourced communities 3. Food insecurity	**D. Individual** **D.1. Genetic factors** a. Monogenetic syndromes and polygenetic effects b. Epigenetic effects **D.2. Prenatal risk** a. Parental obesity b. Maternal weight gain c. Gestational diabetes d. Maternal smoking **D.3. Postnatal risk** a. Birth weight b. Early breastfeeding cessation and formula feeding c. Rapid weight gain during infancy and early childhood d. Early use of antibiotics **D.4. Childhood risk** a. Endocrine disorders b. Special health care needs c. Autism spectrum disorder d. Developmental and physical disabilities e. Myelomeningocele f. Attention-deficit/hyperactivity disorder g. Weight-promoting appetitive traits h. Use of weight-promoting medications i. Depression
**B. Neighbourhood and community** 1. School environment 2. Lack of fresh food access 3. Fast food proximity 4. Access to safe physical activity 5. Environmental health	
**C. Family and home environment** 1. Parenting feeding style 2. Sugar-sweetened beverages 3. Portion sizes 4. Snacking behaviour 5. Dining out and family meals 6. Screen time 7. Sedentary behaviours 8. Sleep duration 9. Environmental smoke exposure 10. Psychosocial stress 11. Adverse childhood experiences	

Motivational interviews and therapeutic education are approaches with good evidence of effectiveness, known to healthcare professionals, especially in primary care, but applying them for obesity is more difficult, requiring more attention, skill and empathy. The stigmatization of weight has made healthcare professionals unwittingly blaming and deriding, and patients, repeatedly offended, have become hypersensitive.

To change care we must accept patients as equal co-authors in care projects, as already done in other countries for diseases, such as diabetes mellitus and obesity.

Without this step, dealing more with obesity could have negative effects. Teaching primary school overweight children, the “mistakes” that occur daily in their families and the complications of excess weight can increase the sense of guilt. Focusing attention on food, weight and body image, favours low self-esteem and eating disorders, which are already frequent [12].

The latest study by Rebecca Puhl’s group [13, 14], who has been dealing with weight stigma for years, on thousands of questionnaires filled out by parents, teenagers and now also children from various countries around the world, has highlighted the role of stigma on both physical and psychological health, and its devastating effect, if started in childhood. We have discovered that in all families, where members meet mainly for shopping, preparing and eating meals, trying on and buying clothing, the discussion about weight is continuous, especially between parents and overweight children and it usually has negative and harmful tones. In order to improve this situation, a guide will soon be published on the website of the Italian Society of Paediatrics to help parents talk to their children and support them during treatment. Furthermore, the Emilia Romagna Region has developed the BeBa (Benessere Bambino, Childhood Wellbeing) app for families to promote healthy childhood initiatives [15].

Today, paediatricians are asked to create guides to help parents give positive messages about weight and body to their children, or at least to avoid talking about it to protect their psychological health and allow the construction of a positive social identity. These guides are necessary for understanding what to do, instead of just talking about it without doing anything, since talking promotes shame and sadness. But how can paediatricians help parents if they themselves use negative messages?

Instead of listening to them and helping them find personalized treatment paths, they load them with guilt and judgement. The list of tips for changing stigma in the healthcare sector is extensive, but still struggles to be adopted (Table 3) [5]. Guides for parents on how to talk about weight and what to do to reduce stigma and improve family lifestyle are necessary.

**Table 3:** Strategies for minimizing weight stigma in healthcare. (adapted from Lister et al. 2023) [5]

**Education of health-care professionals**	
• Know their implicit and explicit weight stigma • Coverage of the broader determinants of obesity focusing on the genetic and/or socioenvironmental determinants of weight.	• Provide empathy-invoking interventions, emphasizing size acceptance, respect and human dignity. • Offer opportunities to practise non-stigmatizing care
• Discussion of harms caused by social and cultural norms and messages concerning body weight	• Provide a weight-inclusive approach, by emphasizing that all individuals, regardless of size, have the right to equal health care.
**Health Facility infrastructure and processes**	
• Provide appropriately sized chairs, blood pressure cuffs, weight scales, beds, toilets, showers and gowns.	• Use non-stigmatizing language in signage, descriptions of clinical services and other documentation.
**Clinical Practice**	
• Providing clinical leadership and using appropriate language within healthcare settings • Senior clinicians and managers should role-model supportive and non-biased behaviours towards people with obesity and not tolerate weight-based discrimination in any form.	• Staff should identify the language that individuals prefer in referring to obesity. • Use person-first language, for example a ‘person with obesity’ rather than ‘obese person’

Luckily not everything is stalled. Our project to collect questionnaires from parents of children and adolescents with overweight/obesity, started with a group of pediatricians from the Society of Paediatric Endocrinology and Diabetology, and welcomed by 17 Level II centers and 10 Family Paediatricians from the Campania Region, has terminated. Parents and children rated on a 5 Likert scale how motivating and offensive they considered 10 terms commonly used by professionals to talk about weight: *weight, excessive weight, unhealthy weight, overweight, obesity, severe obesity, serious obesity, complicated obesity, fat, very fat* [16].

Questionnaires of 391 parents and 249 children (range 5–18 years; average age 11), filled out from June 2019 to February 2020, demonstrate that the term “unhealthy weight” is the most motivating and least offensive. The most offensive terms are: obesity and fat. There is no single term that is valid for everyone, so the only practical advice for healthcare professionals is to use the terminology used by the family or ask them. Terms like a*bove normal weight, inadequate weight, very robust, unhealthy weight*, were the most motivating.

### Network

For years, some family pediatricians and specialists have tried to treat individual patients, but without a proper network, satisfactory and long-lasting results can rarely be achieved with such a disease. The network is essential! Today there is a swarm of small prevention projects that are not coordinated with each other, often sponsored by the food or marketing industry, carried out with small groups and limited timescales, which in the end will not clarify the questions that experts are asking today about how to contrast obesity.

Currently in 11 regions, led by Emilia Romagna, a project is underway, supported by the National Center for Disease Prevention and Control (CCM) 2020 of the Ministry of Health, which re-evaluates the positive contents of past projects and seeks to reactivate them, improve them, put them online and spread them to other institutions among Primary Care, Hygiene and Nutrition Services and specialists. A multi-disciplinary and multi-component family-centered therapeutic project, based on the principles of therapeutic education, as supported by all most recent guidelines, has been active in Emilia Romagna for ten years.

Family paediatricians, which are the strong point not only in the prevention, but above all in the treatment of obesity, adequately trained in their central role (Table 4), are enthusiastic. However, it is necessary to seriously invest in the care of this disease, in order to improve the path, and offer intensive projects with teams trained on social determinants and early adverse events, as proposed by the AAP [2], without forgetting the transition to adult care.

**Table 4:** Role of the Pediatric Health Care Providers. (adapted from Hampl et al. 2023) [2]

**Diagnosis measurement**	• Measure height and weight, calculate BMI and assess BMI Percentile • Communicate BMI and weight status to patient and family
**Evaluation Risk factors**	• Assess individual, structural, and contextual risk factors • Perform comprehensive patient history • Conduct physical exam • Evaluate for comorbidities and order relevant diagnostic studies and laboratories • Assess readiness to change
**Treatment**	• Treat obesity and comorbidities concurrently • Manage children with overweight/obesity following principles of chronic care model and medical home • Deliver non-stigmatizing care • Use Motivational Counselling to engage patient and families in addressing overweight / obesity, set goals and promote participation or utilization of local resources or programs • Promptly engage and refer children to intensive treatment, if available. If this treatment is not available in your area, deliver highest intensity treatment possible as medical home, coordinate care, advocate for family, and support transition to adult care • Address the social determinants of the disease and take care of any early adverse events detected • Offer weight loss pharmacotherapy, to eligible patients, according to medication indications, risks, and benefits, as an adjunct • For eligible patients with severe obesity, offer referral to a local or regional comprehensive multidisciplinary paediatric metabolic and bariatric surgery center for evaluation.

## Conclusions

It will take time to see a change everywhere, but the most difficult first step, which is changing the narrative, does not require huge economic investments, but rather personal growth paths for professionals: with adequate training in the use of terms in healthcare to send messages of “RESPECT”. Thus, we can begin to improve the therapeutic approach and the outcome of treatment, reduce the internalization of weight stigma, prevalence of drop-out and change the future history of children with excess weight. The next step will be to spread the process everywhere, exploiting all possible resources in the network. In short, there still is hope!

## Data Availability

Not applicable.

## Abbreviations

BMI: Body Mass Index
WHO: World Health Organization
CCM: National Center for Disease Prevention and Control

## References

[1] Tanas R, Bernasconi S, Marsella M, Corsello G (2020). What’s the name: the weight stigma and the battle against obesity. Ital J Pediatr.

[2] Hampl SE, Hassink SG, Skinner AC, Armstrong SC, Barlow SE, Bolling CF (2023). Clinical practice Guideline for the evaluation and treatment of children and adolescents with obesity. Pediatrics.

[3] Hadjiyannakis S, Buchholz A, Chanoine JP, Jetha MM, Gaboury L, Hamilton J (2016). The Edmonton obesity staging system for pediatrics: a proposed clinical staging system for paediatric obesity. Paediatr Child Health.

[4] WHO European Regional Obesity Report. 2022. Copenhagen: WHO Regional Office for Europe; 2022. Licence: CC BY-NC-SA 3.0 IGO. (https://iris.who.int/bitstream/handle/10665/353747/9789289057738-eng.pdf?sequence=1)

[5] Lister NB, Baur LA, Felix JF, Hill AJ, Marcus C, Reinehr T (2023). Child and adolescent obesity. Nat Rev Dis Primers.

[6] Puhl RM, Lessard LM, Foster GD, Cardel MI (2023). Parent-child communication about weight: priorities for parental education and support. Pediatr Obes.

[7] Hoeeg D, Frohlich KL, Christensen U, Grabowski D (2023). Mechanisms of stigmatization in family-based Prevention and Treatment of Childhood overweight and obesity. Children.

[8] Lee KM, Arriola-Sanchez L, Lumeng JC, Gearhardt A, Tomiyama AJ (2022). Weight Stigma by Association among Parents of Children with Obesity: a Randomized Trial. Acad Pediatr.

[9] Braddock A, Browne NT, Houser M, Blair G, Williams DR (2023). Weight stigma and bias: a guide for pediatric clinicians. Obes Pillars.

[10] Halford JCG, Bereket A, Bin-Abbas B, Chen W, Fernández-Aranda F, Garibay Nieto N (2022). Misalignment among adolescents living with obesity, caregivers, and healthcare professionals: ACTION teens global survey study. Pediatr Obes.

[11] English S, Vallis M (2023). Moving beyond eat less, move more using willpower: reframing obesity as a chronic disease impact of the 2020 Canadian obesity guidelines reframed narrative on perceptions of self and the patient-provider relationship. Clin Obes.

[12] Jebeile H, McMaster CM, Johnson BJ, Garnett SP, Paxton SJ, Seidler AL (2023). Identifying factors which influence eating disorder risk during behavioral Weight Management: a Consensus Study. Nutrients.

[13] Lawrence SE, Puhl RM, Watson RJ, Schwartz MB, Lessard LM, Foster GD (2023). Family-based weight stigma and psychosocial health: a multinational comparison. Obes (Silver Spring).

[14] Lessard LM, Puhl RM, Foster GD, Cardel MI (2023). Parental communication about Body Weight and Adolescent Health: the role of positive and negative weight-related comments. J Pediatr Psychol.

[15] Tanas R, Licenziati MR, Calcaterra V, Corica D, Morino G, Caruso M et al. Quali parole per curare l’obesità in età evolutiva? [What words to cure obesity in developmental age?] Rivista Italiana di Pediatria Preventiva e Sociale (Parma). 2021;16(1):32–6. https://www.sipps.it/attivita-editoriale/ripps-rivista/anno-xvinumero-1/

[16] Bonvicini L, Davoli AM, Ferrari E, Ilari B, Compiani M, Patrignani N (2022). Un’esperienza di mobile health nell’AUSL Di Reggio Emilia: dalla co-creazione alla co-valutazione dell’app BeBa per la prevenzione dell’obesità infantile E La Promozione Di Sani Stili Di Vita Nei Bambini. Boll Epidemiol Naz.

